# Acceptance of Teledermoscopy by General Practitioners and Dermatologists in Denmark

**DOI:** 10.5826/dpc.1102a33

**Published:** 2021-04-12

**Authors:** Tine Vestergaard, Merethe K. Andersen, Anette Bygum

**Affiliations:** 1 Department of Dermatology and Allergy Centre & Odense Patient Data Explorative Network, Odense University Hospital, Odense, Denmark; 2 Audit Project Odense, Research Unit of General Practice, University of Southern Denmark, Odense, Denmark; 3 Department of Dermatology and Allergy Centre, Odense University Hospital, Odense, Denmark

**Keywords:** provider satisfaction, teledermoscopy, teledermatology, skin cancer, malignant melanoma, general practitioner

## Abstract

**Background:**

Teledermoscopy can be used to triage referrals of suspected skin cancers, thereby reducing waiting time and number of face-to-face consultations with a dermatologist. However, the success of the implementation of this technology in part relies on the acceptance of the providers.

**Objectives:**

This study assessed the attitudes towards teledermoscopy of referring general practitioners and consultant dermatologists.

**Methods:**

General practitioners from 48 practices and 3 dermatologists in the region of Southern Denmark, who had previous experience with teledermoscopy, were invited to answer questionnaires on their acceptance of the technology.

**Results:**

General practitioners from 23 practices responded. All domains of the questionnaire received high scores, indicating a high degree of acceptance of teledermoscopy among respondents. All 3 dermatologists agreed that teledermoscopy was useful for triaging referrals, but they were less confident in their diagnoses and management plans proposed by teledermoscopy than in traditional face-to-face evaluations of patients. Two of the 3 dermatologists were satisfied with using teledermoscopy as a consult method.

**Conclusions:**

This study reports high levels of provider acceptance of teledermoscopy. However, a low response rate among general practitioners may limit its generalizability.

## Introduction

The rising incidence of skin cancer affects the lives of many and poses a burden to health care systems across the globe [1,2]. In Denmark, persons who suspect they might have skin cancer usually consult their general practitioner (GP), who acts as a gatekeeper in triaging these lesions for further evaluation by a dermatologist or plastic surgeon. However, early signs of skin cancer may be subtle, and a sensitivity of 50%–55% for the detection of malignant melanoma has been reported for GPs [3]. Telemedicine is useful in dermatology, a medical specialty that relies on visual inspection and pattern recognition [4]. However, telemedicine is currently not recommended in Denmark for the evaluation of possible skin cancers. In the examination of skin tumors, dermatologists use dermoscopy, which has been shown to increase the diagnostic sensitivity and specificity [5]. Given the right equipment, GPs can take dermoscopic images and forward them for evaluation by a dermatologist. This has been reported to be useful as a triage tool and can reduce waiting times [6].

We previously studied the diagnostic accuracy of teledermatology, including teledermoscopy, in the region of Southern Denmark in 2018 [7]. In the current study, we assessed the acceptance of this new technology among participating GPs. In addition, we collected the views of the participating dermatologists. Satisfaction of both patient and provider is believed to be an important factor in the successful implementation of novel technologies [8,9]. We previously reported that almost 90% of patients were satisfied with, or neutral towards, the use of teledermoscopy [10].

## Materials and Methods

### Prior Diagnostic Accuracy Study

GPs from 50 practices in the region of Southern Denmark had been included in the diagnostic accuracy study [7]. Background information on age, sex, years working as a GP, interest in dermatology and dermoscopy, and distance to the nearest dermatologist was gathered in January 2018. During the study, 2 general practices dropped out and 1 general practice did not include any patients. A total of 519 patients with 600 possible skin cancers were included. On average, each practice photographed 12.5 lesions (range, 0–41) with an iPhone and Handyscope, which were sent for evaluation by a dermatologist using the FotoFinder hub [11]. The study was initialized with a teaching session on dermoscopy by the primary investigator in January 2018 and was completed with an evaluation and feedback session in December 2018 as proposed by the APO method [12]. GPs from 29 general practices attended this meeting. In February 2019, we e-mailed an electronic questionnaire on provider acceptance of teledermoscopy to all 48 participating general practices, with a reminder 2 weeks later.

### Questionnaire for GPs

The questionnaire used to assess the GPs’ acceptance of teledermoscopy was the modified Technology Acceptance Model (TeleTAM) developed by Orruño et al [13]. TeleTAM was developed based on existing theories on adaptation and acceptance of new technologies, and was face-and content-validated by experts. It consists of 33 questions exploring 8 domains believed to influence the acceptance of teledermatology by physicians (Table 1). The providers’ future intention to use teledermatology (INT) is the main outcome. The other 7 domains consist of items related to the individual context (compatibility, attitude), the technological context (perceived usefulness, perceived ease of use, habits), and the organizational context (facilitators, subjective norm). At the end of the questionnaire, GPs had the option to comment in free writing.

We translated the English version of the questionnaire into Danish after a forward translation and back-translation process. The word “teledermatology” in the original questionnaire was replaced by “teledermoscopy” in this study. Answers to the questionnaire were given on a 7-point Likert scale ranging from strongly disagree to strongly agree. Questionnaires were created, sent and stored in REDCap (Research Electronic Data Capture) hosted at OPEN (Open Patient data Explorative Network), Odense University Hospital [14,15].

We speculated that a GP’s intention to use teledermoscopy might be influenced by having a dermoscope or a special interest in dermatology before the study, or by the distance to the nearest dermatologist.

### Questionnaire for Dermatologists

Three dermatologists who had participated in the diagnostic accuracy study on teledermoscopy were asked about their views on this novel procedure via a short questionnaire. They had 1, 4 and 9 years of experience post-specialization, and had reviewed 25.8%, 24.8% and 26.2% of the cases referred for teledermoscopy, respectively. A fourth dermatologist, who was an investigator in this study and hence not surveyed, had evaluated the remaining photos.

To evaluate the dermatologists’ perceptions of teledermoscopy, the 5 questions previously applied by Whited and colleagues were used [16]. These questions were considered to have face validity as they treated issues pivotal to the future implementation of teledermoscopy. A paper form with the original English wording and a Danish translation was used. Since the dermatologists evaluated both clinical and dermoscopic images of each skin lesion, the word “teledermatology” was maintained.

## Statistical Analysis

STATA version 16.0 was used for all statistical methods. P values <.05 were considered statistically significant. In concordance with the study by Orruño et al [13], a score was calculated for each domain as the mean of the scores of the questions related to that domain. For the logistic regression analysis, the dependent variable (INT) was dichotomized into 0 = low/moderate and 1 = high intention to use teledermoscopy. As in the study by Orruño et al [13], we chose the median as the cut-off point between the groups; a score ≥6 was rated as high intention.

Because only 29 observations were available, the number of explanatory variables needed to be reduced for the regression analyses. Based on previous studies, 3 domains, namely perceived usefulness (PU), perceived ease of use (PEU) and facilitators (FAC), were considered most important [13,17]. Orruño et al [13] only found FAC to be a significant predictor of INT. PU and PEU were dimensions in the original Technology Acceptance Model proposed by Davis [18], and both domains had high correlations with INT. We found collinearity between the variables PU and PEU and decided to reduce our statistical model further by excluding PEU.

Cronbach alpha and inter-item correlation were calculated to elucidate the reliability and validity of the TeleTAM questionnaire.

## Results

### General Practitioners’ Responses

Twenty-nine GPs from 23 different practices completed the TeleTAM questionnaire. GPs from another two practices reporting on technical problems did not respond, for a total of 68 non-respondents. Background information on respondents and non-respondents, collected during the diagnostic accuracy study, is shown in Table 2. Respondents were significantly older than non-respondents and there was a trend towards respondents being more interested in dermatology. Scores on the TeleTAM questionnaire, for the 8 domains, and Cronbach alpha and inter-item correlation are shown in Table 3. All domains received high scores, indicating a high degree of acceptance of teledermoscopy among respondents. Cronbach alpha was acceptably high for all variables except FAC and COM. Correlation with INT was moderate to high for all domains except HAB.

Logistic regression with a very reduced model due to the low number of observations did not reveal PU or FAC as significant predictors of INT. Having a special interest in dermatology and the distance to the nearest dermatologist did not significantly predict high INT. Interestingly, GPs who did not own a dermoscope were 10-times more likely to report high INT (95% CI, 1.3–78.1, P = .03).

A few comments in free writing were received. Two GPs stressed the lack of reimbursement as a disadvantage, and one mentioned the cost of the equipment as a barrier. One GP commented that he still lacked routine in dermoscopy. One GP commented on the questionnaire and wanted the option to answer “not relevant.” One GP commented that he had invested in a Handyscope.

### Dermatologists’ Responses

**Figure 1:**
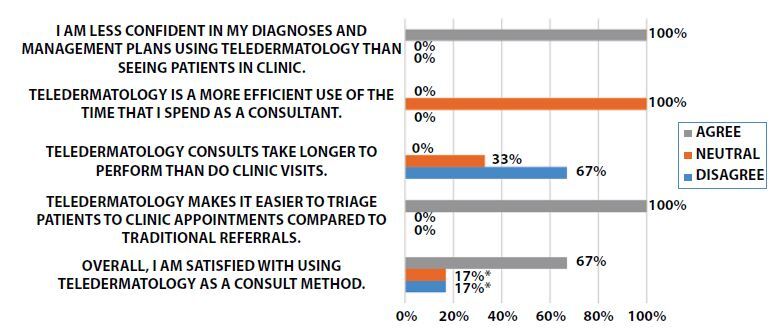
Dermatologists’ views on teledermoscopy (n = 3). *One dermatologist answered both neutral and disagree.

The results of the questionnaire for dermatologists are shown in Figure 1. All 3 dermatologists agreed that teledermoscopy was useful for triaging referrals, but they were less confident in their teledermoscopy diagnoses and management plans than in the traditional face-to-face evaluations of patients. Two of the 3 dermatologists were satisfied with teledermoscopy as a consult method.

## Discussion

Nowadays, in the era of social distancing, the interest in teledermoscopy is definitely increasing, not only among dermatologists but also among GPs. Together, these healthcare providers proved to easily use a common web platform for teledermoscopy and research purposes [19,20]. In this questionnaire study, Danish GPs were asked about their attitudes towards the use of teledermoscopy. Overall, there was a very positive attitude towards this new technology, with mean scores for all domains above what has previously been reported on the TeleTAM questionnaire [13,17]. While high levels of satisfaction with teledermatology have been reported for both primary care physicians and dermatologists, only a few studies specifically evaluated teledermoscopy [21]. Kenney et al found high levels of satisfaction with teledermoscopy in a survey of 5 primary care physicians in a hospital setting, where a trained nurse photographer took the photos [22]. In a study of mixed quantitative and qualitative measures of provider satisfaction with teledermoscopy, Janda et al concluded that most participants were receptive to the use of mobile teledermoscopy in their practice [23]. Both GPs and dermatologists were surveyed. The study elucidated advantages and disadvantages of teledermoscopy and touched on themes such as lesion monitoring, time consumption and record keeping, which we recognize from the comments in the TeleTAM questionnaire and feedback obtained at the evaluation session.

Orruño et al found FAC to be the only significant predictor of INT [13]. We could not replicate this result, possibly due to the low number of observations. However, we found the lowest Cronbach alpha for FAC, indicating that the reliability of this domain may be low in different health care settings. We also found low reliability for COM, as did Stratton and Loescher [17]. Furthermore, Stratton and Loescher reported low reliability for HAB. In our study, the Cronbach alpha for this domain was acceptably high. The validity of the TeleTAM questionnaire seems good. We found the lowest correlation with INT for HAB; this corresponds to the findings of both Orruño et al [13] and Stratton and Loescher [17].

We found that GPs without a dermoscope were more likely to have high intentions to use teledermoscopy. This may be because GPs already using dermoscopy have higher confidence in their diagnoses and have less need for specialist assistance through teledermoscopy or a standard referral, as shown by Chappuis et al [24]. Another study reported that 82% of GPs using dermoscopy were confident or very confident in its use [25]. The distance to the nearest dermatologist did not influence INT. However, distances to dermatologist are relatively short in all of Denmark compared to Australia and some Nordic countries, where teledermoscopy may be even more warranted than in Denmark.

A major limitation to this study is the low number of observations. GPs have limited time and an increasing workload; we speculate that this may be an explanation for the low participation rate. Other studies reported response rates of 2%, 62% and 100% in different set-ups, with the highest response rate reported in a study of 5 primary care physicians in a hospital setting [13,22,26]. Furthermore, respondents and non-respondents in our study differed in several ways. Firstly, respondents were older than non-respondents. The background information was collected at the beginning of the diagnostic accuracy study in January 2018. More than 90% of the general practices employed GPs in training (data not shown). In Denmark, this type of employment lasts 6 or 12 months. Therefore, many of these young doctors, who had potentially participated in the diagnostic accuracy study, were no longer employed at the participating practices at the time of this questionnaire study and hence could not answer the TeleTAM questionnaire. This may also in part explain the low number of respondents. In addition, more respondents had a special interest in dermatology, which may bias their attitude towards teledermoscopy. Therefore, our results may not be generalizable, and Danish GPs as a whole may not be as positively minded towards this new technology. However, as some of the barriers towards the use of teledermoscopy may be lacking remunerations, this could be an incentive worth taking into account in future collective bargaining. Finally, our results may not be directly transferable to primary care settings in other parts of the world. In conclusion, this study found a high acceptance of teledermoscopy among GPs and dermatologists. More studies investigating provider acceptance and satisfaction with teledermoscopy are warranted.

## Figures and Tables

**Table 1:** The 8 Domains of the TeleTAM Questionnaire

Domain	Example question^a^
PU: perceived usefulness	Teledermoscopy could help me to diagnose my patients more rapidly.
PEU: perceived ease of use	I think that teledermoscopy is a flexible technology to interact with.
FAC: facilitators	I would use teledermoscopy if I receive adequate training.
COM: compatibility	The use of teledermoscopy is compatible with my work habits.
SN: subjective norm	Most of my colleagues will welcome the fact that I use teledermoscopy.
HAB: habits	I feel comfortable with information and communication technologies.
ATT: attitude	The use of teledermoscopy is beneficial for the diagnosis of my patients.
INT: intention to use	I have the intention to use teledermoscopy routinely with my patients.

a The questionnaire comprises 33 questions overall. One example question is shown for each domain.

**Table 2:** Background Information on Participating GPs, According to Whether or Not They Responded to the TeleTAM Questionnaire^a^

Characteristic	Respondents (n=29)		Non-respondents (n=68)		P^b^
	No.^a^	Value	No.^a^	Value	
Male sex, n (%)	28	18 (64)	68	31 (46)	.10
Age, mean (SD), y	24	50.7 (8.6)	68	46.2 (10.6)	.04
Years working as GP, mean (SD)	22	13.7 (9.4)	63	9.7 (9.3)	.10
Special interest in dermatology	22	14(63.6)	61	25 (41.0)	.07
Have a dermoscope	23	11(47.8)	62	28 (45.2)	.83
Distance to nearest dermatologist, mean (SD), km	23	9.8 (11.2)	64	9.3 (11.2)	.85

a Number of persons who provided the requested information;

b P values calculated by two-sample *t* test with unequal variance.

GP = general practitioner; SD = standard deviation.

**Table 3:** GPs’ Scores on the TeleTAM Questionnaire, and Crohnbach Alpha and Correlation with Intention to use Teledermoscopy

Variable	Observations, n	Score, Mean (SD)	Minimum Score^a^	Cronbach alpha	Correlation with INT
PU	28	6.10 (0.65)	4.83	0.91	0.80
PEU	29	5.75 (0.80)	4.50	0.89	0.65
FAC	29	6.03 (0.57)	4.67	0.35	0.67
COM	29	5.15 (0.63)	3.75	0.52	0.53
SN	28	5.71 (0.84)	4.00	0.84	0.63
HAB	29	5.84 (0.96)	4.00	0.71	0.38
ATT	29	6.15 (0.57)	5.00	0.80	0.71
INT	28	6.07 (0.77)	4.33	0.90	1.00

a The maximum score for every variable was 7.00.
